# Polymer Materials for High‐Performance Triboelectric Nanogenerators

**DOI:** 10.1002/advs.202000186

**Published:** 2020-06-02

**Authors:** Aihua Chen, Chen Zhang, Guang Zhu, Zhong Lin Wang

**Affiliations:** ^1^ School of Materials Science and Engineering Beihang University Beijing 100191 P. R. China; ^2^ CAS Center for Excellence in Nanoscience Beijing Key Laboratory of Micro‐nano Energy and Sensor Beijing Institute of Nanoenergy and Nanosystems Chinese Academy of Sciences Beijing 100083 P. R. China; ^3^ New Materials Institute Department of Mechanical, Materials and Manufacturing Engineering University of Nottingham Ningbo China Ningbo 315100 P. R. China; ^4^ School of Materials Science and Engineering Georgia Institute of Technology Atlanta GA 30332 USA

**Keywords:** diverse energy harvesting, energy conversion technologies, polymer materials, triboelectric nanogenerators

## Abstract

As an emerging branch of energy conversion technologies, the triboelectric nanogenerator (TENG) pioneers a brand‐new path to effectively harness varieties of mechanical energies for the purpose of powering and/or sensing. Since its invention in 2012, the TENG has experienced a booming and revolutionary development in every respect, ranging from materials synthesis and modification, architecture design to performance optimization, power management, and application exploration. In comparison to the organic solar cell and organic light‐emitting diodes, TENG is a unique technique that opens the venue of using polymer materials (PMs) for harvesting mechanical energy. So far, by virtue of superior charge transfer and capturing capabilities during friction, various kinds of PMs have been developed and used as triboelectric materials in order to achieve high‐performance TENGs. Here, this work focuses on the utilization and development of PMs for the TENGs technology and first gives a summary of main PMs that are frequently adopted in currently reported energy‐harvesting TENGs. Second, several kinds of PMs used lately in a few novel TENGs for special or specific energy‐harvesting circumstances are introduced and highlighted. Finally, the perspectives on and challenges in developing high‐performance PMs toward TENGs technology are conceived and expected to be instructive to future research.

## Graphical abstract

As an emerging energy conversion technology, the triboelectric nanogenerator (TENG) opens a venue of using polymer materials (PMs) for harvesting mechanical energy. This work summarizes the main PMs in currently reported TENGs, and introduces several PMs used in a few novel special TENGs. Meanwhile, the perspectives on developing high‐performance PMs toward TENGs are conceived and expected to be instructive to future research.

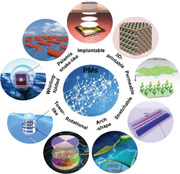

## 1 Introduction

**Figure 1:**
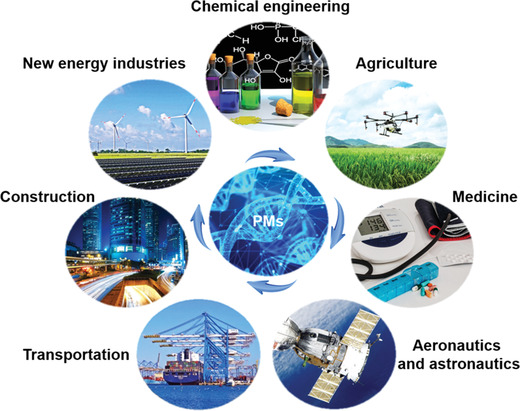
Schematic diagram of various application fields of the PMs.

In the long history of mankind, materials have always been the cornerstone of human society development, even from Palaeolithic age up to now. Without exaggeration, they can reach every corner and cover almost all aspects in our daily life, such as dressing, dining, settling, and traveling. Among them, polymer materials (PMs) are of particular significance since artificially synthesized PMs appeared in 1830s, by virtue of their special mechanical, physicochemical, and other unique properties compared to classic metal and inorganic materials.^[^ ^1^, ^2^ ^]^ Now the PMs, as graphically presented in **Figure** **1**, have already been widely and deeply used in a great many fields that encompass chemical engineering, agriculture, medicine, aeronautics and astronautics, transportation, construction, and new energy industries, among others. Specifically, PMs can play various key roles in these fields, including, but not limited to, plastic and rubber products, decorative coatings, matrix of composites, biological medicines, and active substances for kinds of physical/chemical reactions.^[^ ^3^, ^4^, ^5^, ^6^, ^7^, ^8^, ^9^ ^]^ In particular, the development and utilization of PMs in energy industry have been prominently accelerated and extended over the past decades, due to unprecedented advances in a series of technical innovations of energy conversion toward diverse renewable energy resources (solar, geothermal, wind, tide energy, etc.).^[^ ^10^, ^11^, ^12^ ^]^

**Figure 2:**
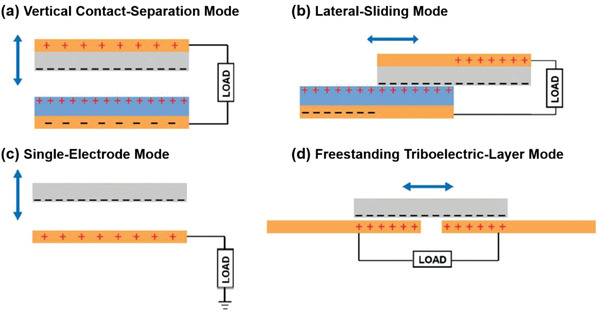
Four working modes of TENGs. a) Vertical contact‐separation mode. b) Lateral‐sliding mode. c) Single‐electrode mode. d) Freestanding triboelectric‐layer mode. a–d) Reproduced with permission.^[^ ^17^ ^]^ Copyright 2019, Wiley‐VCH.

**Figure 3:**
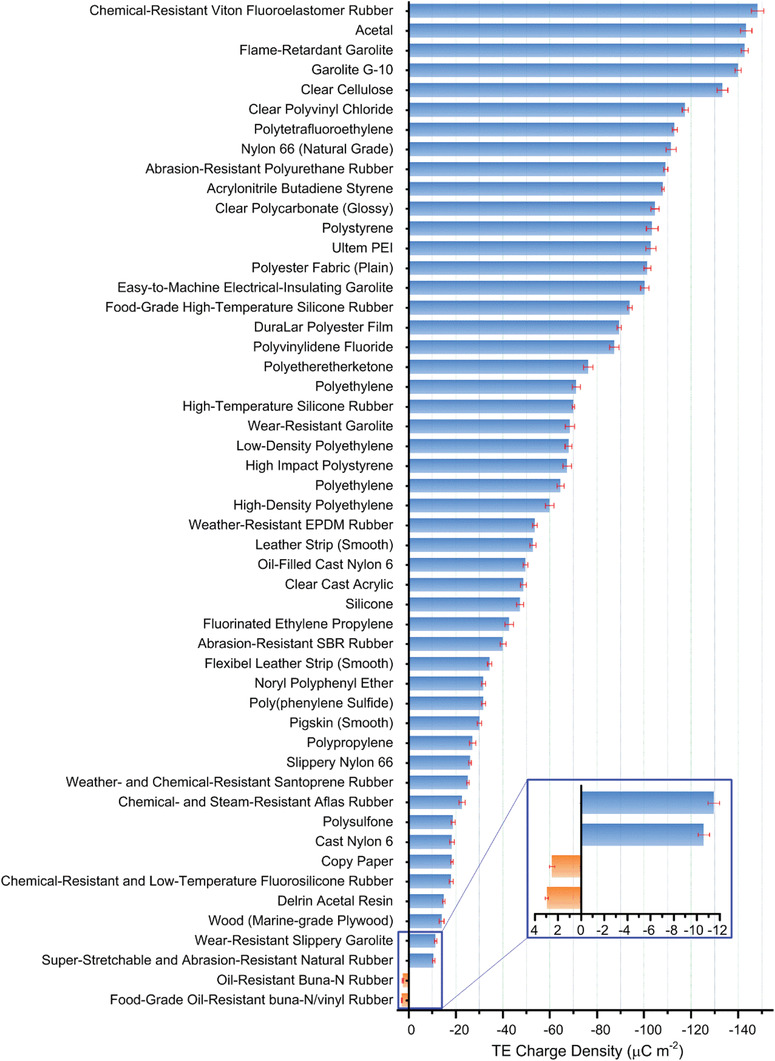
The quantified triboelectric series measured by contacting with mercury. The error bar indicates the range within a standard deviation. Reproduced with permission.^[^ ^32^ ^]^ Copyright 2019, Springer Nature.

As an emerging branch of energy conversion technologies, triboelectric nanogenerator (TENG) invented in 2012 opens up a brand new path for effectively harnessing varieties of mechanical energies,^[^ ^13^ ^]^ which are ubiquitous and abundant but usually wasted in our ambient environment. So far, the TENG has experienced a rapid and booming development period, ranging from architecture design, materials selection, and modification to performance optimization, power management, and application exploration.^[^ ^14^, ^15^, ^16^, ^17^, ^18^, ^19^, ^20^, ^21^, ^22^, ^23^, ^24^, ^25^ ^]^ By coupling two common phenomena of contact‐electrification effect and electrostatic induction, four types of working modes have been gradually evolved and proposed for TENGs, as shown in **Figure** **2**.^[^ ^17^ ^]^ In all cases, oppositely polarized triboelectric charges can be generated on material's surfaces during the contact‐electrification process and then the electrostatic induction renders a driving force for transformation of mechanical stimuli into electric energy when relative motion occurs. Based on fundamental physical model of Maxwell's displacement current, the TENGs can be essentially regarded as a kind of capacitive variable electric‐field source, and their output power is proportional to the square of triboelectric charge density.^[^ ^26^, ^27^ ^]^ That is to say, the key to improve TENGs’ performance is trying to substantially increase the amount of generated triboelectric charges. In principle, the greater the difference of electron affinity between two triboelectric materials is, the more the triboelectric charges can be generated.^[^ ^17^, ^25^ ^]^ **Figure** **3** provides a triboelectric series for numerous materials ranked according to their capabilities of gaining or losing electrons against friction, which can guide researchers to obtain high output performance for TENGs from the triboelectric material selection point of view.^[^ ^28^, ^29^, ^30^, ^31^, ^32^ ^]^ Note that the majority of materials in the list belong to PMs, which possess all sorts of functional groups, such as fluorine (—F), cyano group (—CN), ester group (—COOR), acyl group (—CON—), carboxyl (—COOH), and nitro (—NO_2_) as electron‐withdrawing groups, and amidogen (—NH_2_), amide group (—CONH), oxhydryl (—OH), and alkoxy (—OR) as electron‐donating groups. All of these functional groups can play a major role in charge transfer and charge capturing during contact‐electrification process by right of their unique hybrid orbital configurations. Moreover, featuring other merits of superior flexibility, machinability, stretchability, scalability, and low weight, PMs have thus inevitably become the core foundation of the TENGs technology.

Now the TENGs have been developed with revolutionary applications mainly in four fields: micro/nanopower sources, large‐scale blue energy harvesting, self‐powered active sensors, and direct high voltage (HV) sources for specific systems. Several reviews have summarized the progress from each aspect, including theoretical modeling,^[^ ^17^ ^]^ energy harvesting,^[^ ^13^, ^16^, ^18^, ^20^, ^33^ ^]^ active sensing,^[^ ^15^, ^19^, ^24^ ^]^ and HV applications.^[^ ^22^, ^23^ ^]^ In all of these fields, the TENGs are expected to yield output performance as high as possible for achieving more efficient energy conversion or higher sensory sensitivities. In order to push ahead with improvement of TENGs’ performance, one of challenges that was proposed by Wang in 2014 is related to high‐performance triboelectric materials with the highest surface charge density.^[^ ^34^ ^]^ Over the past few years, substantial efforts have been made to investigate high‐performance triboelectric materials for diverse energy harvesting via a series of methods, including exploration of novel PMs, physical/chemical modification of PMs, and material surface engineering.^[^ ^25^ ^]^ Here, this review focuses on the utilization and development of PMs for TENGs technology and first gives a summary of main PMs that are frequently adopted in currently reported energy‐harvesting TENGs. Second, several kinds of novel PMs developed and used recently in TENGs for special or specific energy‐harvesting circumstances are introduced and highlighted. Finally, key priorities of research challenges and directions for PMs’ development toward high‐performance TENGs are conceived and expected to be instructive to future research works.

## 2 Main PMs Adopted in Currently Reported TENGs

Plenty of materials have been used to fabricate TENGs since its invention, due to the fact that almost all materials possess triboelectrification effect, from metal, to wool, to wood, and to PMs.^[^ ^25^ ^]^ Although optional pairs of triboelectric materials are countless, not every pair can bring in high output performance. As expounded herein before, the output of TENGs is primarily dependent on the electron affinity difference of the triboelectric material pairs. Therefore, PMs that contain fluorine, such as polytetrafluoroethylene (PTFE), fluorinated ethylene propylene (FEP), and polyvinylidene fluoride (PVDF), have always been used as electron‐withdrawing part of the TENGs owing to the strongest electron attractive force of fluorine element. In turn, PMs containing electron‐donating groups, such as nylon, silk, and wool, have been frequently used as electrophobic part of the TENGs. In the following, main types of PMs that adopted in currently developed TENGs are summarized based on several recent progress reports and their applications in diverse energy harvesting are also described.

### 2.1 PTFE‐Based TENGs

**Figure 4:**
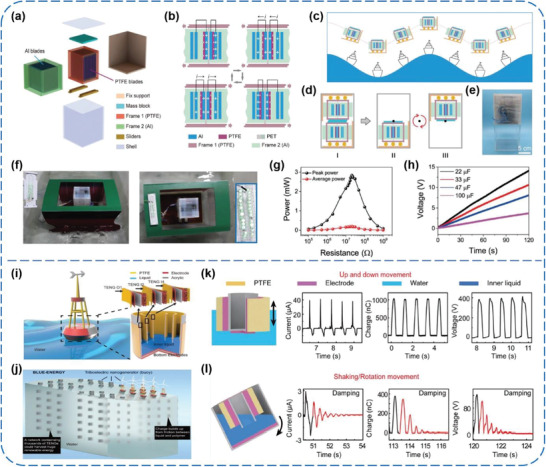
a–h) Tilting‐sensitive TENGs for energy harvesting from unstable/fluctuating surfaces. Reproduced with permission.^[^ ^35^ ^]^ Copyright 2019, Wiley‐VCH. i–l) Buoy‐like liquid–solid interface contact‐electrification‐based TENGs for harvesting low‐frequency blue energy. Reproduced with permission.^[^ ^36^ ^]^ Copyright 2018, Wiley‐VCH.

It is well‐known that PTFE is the most common electronegative material at the current stage and has thus been utilized in the majority of cases for TENGs’ fabrication.^[^ ^35^, ^36^, ^37^, ^38^, ^39^, ^40^, ^41^, ^42^, ^43^, ^44^, ^45^, ^46^ ^]^ As shown in **Figure** **4**a, Zhong et al. reported a tilting‐sensitive TENG (TS‐TENG) that can effectively harness sway energy from some unstable or fluctuating surfaces of ships.^[^ ^35^ ^]^ The TENG contains a slidable frame with 14 PTFE blades inside and a stationary frame with 15 aluminum (Al) blades inside. These two blade structures intersect each other, and as a result, 28 fundamental TENG units are formed in one TS‐TENG. After structural optimization (Figure 4c–e), the transferred charges (half‐cycle) of 2.3 µC, peak current of 25.97 µA, and output voltage of 302.87 V could be generated after rectification for the TENG agitated at a wave frequency of 1.2 Hz and an agitation amplitude of 2.5 V. In addition, a peak power of 2.84 mW and an average power of 0.2 mW were obtained, respectively, with the optimal matching resistance around 20.6 MΩ (Figure 4g). To intuitively show the electricity generation from water wave, the TS‐TENG was utilized to power 30 LEDs and charge different capacitors successfully (Figure 4f,h), which indicate great potential of the TENG in energy harvesting from unstable or fluctuating surfaces for driving various electronic devices on smart ships. Li et al. demonstrated a buoy‐like high‐performance liquid–solid‐interface TENG (LS TENG) for converting low‐frequency blue energy.^[^ ^36^ ^]^ It is made of PTFE films and metal electrodes (Ag), which are lightweight, low‐cost, physically/chemically stable, and mechanically robust in a sealed space in the ocean (Figure 4i). When the LS TENG moves up and down in the water (Figure 4k), the short‐circuit current (*I*_SC_), transferred charge (*Q*_SC_), and open‐circuit voltage (*V*_OC_) of the outer TENG O1 (8 cm × 10 cm) could reach to 40 µA, 1000 nC, and 400 V respectively, being able to light up nearly 100 LEDs. Under shaking or rotation movement, an *I*_SC_ of 3 µA, a *Q*_SC_ of 400 nC, and a *V*_OC_ of 120 V could be generated by the inner TENGs due to the friction between the PTFE films and inner liquid (Figure 4l). Moreover, the output can be multiplied by integrating numerous LS TENG units to construct a network (Figure 4j). With 18 units electrically connected in parallel, a TENG network could generate outputs of 300 V, 290 µA, and 16 725 nC, which make the network a direct power source to construct self‐powered systems, such as a wireless Save Our Souls (SOS) system for ocean emergencies.

**Figure 5:**
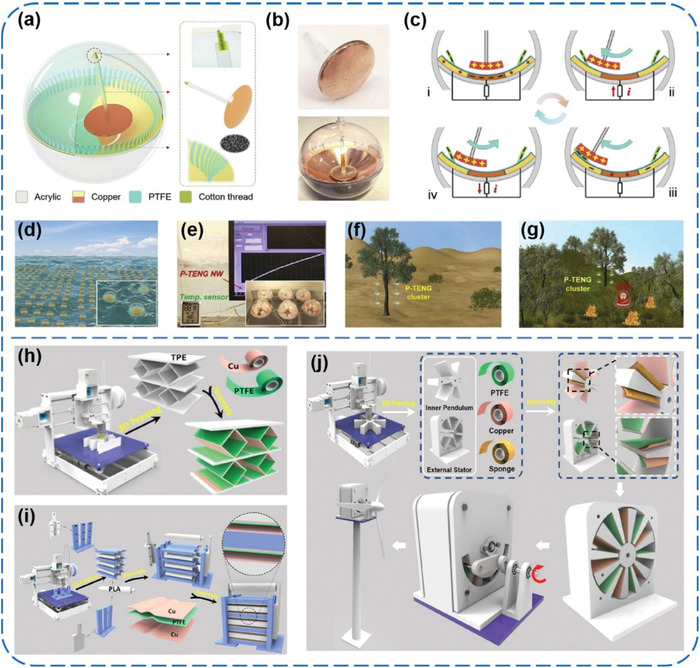
a–g) A super‐robust and frequency‐multiplied pendulum‐inspired TENG for efficient harvesting of water and wind energy. Reproduced with permission.^[^ ^37^ ^]^ Copyright 2019, Elsevier. h–j) Three kinds of 3D‐printed TENGs with different structures for harvesting various mechanical energy. Reproduced with permission.^[^ ^38^ ^]^ Copyright 2019, Elsevier.

Lin et al. developed a pendulum‐inspired TENG (P‐TENG) with multiplied output frequencies for efficiently harnessing water and wind energy.^[^ ^37^ ^]^ The 3D structural scheme of the P‐TENG is shown in **Figure** **5**a, which comprises an electrode layer (Cu), a pendulum triboelectric layer (Cu), and PTFE thin stripes (Figure 5b). Once the P‐TENG is subjected to an external triggering, the pendulum triboelectric layer will relatively swing back and forth and periodically come in contact with the thin PTFE stripes slightly (Figure 5c). After an external excitation, the output performance of the P‐TENG could last 120 s with a frequency of 2 Hz. For harvesting wave energy in a large‐scale, the P‐TENGs were integrated into an array, as schematically shown in Figure 5d. An array with 2 × 3 formation was able to charge a 100 µF capacitor from 0 to 3.1 V within 780 s, and further successfully realized self‐powered temperature measurement (Figure 5e). To scavenge abundant wind energy, a number of P‐TENGs were hung on the tree branches in the desert, as depicted in Figure 5f. Various applications have been explored by utilizing the power harvested from wind energy, such as self‐powered traffic lights and wireless forest fire warning system (Figure 5g). More recently, Gao et al. adopted 3D printing technology to fabricate frames of the TENGs for some personalized demand.^[^ ^38^ ^]^ As shown in Figure 5h‐j, three kinds of TENGs including a printed elastic TENG (PE‐TENG), a series of printed multilayer linkage TENGs (PMLL‐TENG), and a printed multilayer asway TENG (PMLA‐TENG) were fabricated. The triboelectric materials used among all of them were PTFE films and Cu foils. In response to mechanical compression or rotational force, the *V*_OC_ from 410 to 2360 V, *I*_SC_ from 420 µA to 1.7 mA, and the output power density from 1.48 to 6.7 W m^−2^ could be obtained for the three kinds of TENGs, respectively. This work well‐combined 3D printing technology with the manufacture of customized TENGs toward further self‐powered applications. Moreover, Zhu et al. developed a shape‐adaptive micro‐grating‐based TENG (MG‐TENG) made of PTFE thin film with a pair of metal gratings on its opposite sides, which can even convert mechanical sliding motions into electric energy.^[^ ^39^ ^]^ Operating at a sliding velocity of 10 m s^−1^, a MG‐TENG of 60 cm^2^ in overall area, 0.2 cm^3^ in volume, and 0.6 g in weight could deliver an average output power of 3 W (power density of 50 mW cm^−2^ and 15 W cm^−3^) with an overall conversion efficiency of ≈50%, making it a sufficient power supply to regular electronics, such as light bulbs. In addition to commercial films, PTFE in other forms can be also used as triboelectric materials, such as balls, nanofibers, expanded film, and so forth.^[^ ^43^, ^45^, ^46^ ^]^ Zhao et al. prepared a vertical contact‐separation TENG by using an electrospun fibrous PTFE porous membrane and a polyamide‐6 membrane.^[^ ^43^ ^]^ Through a simple negative charge‐injection process onto the PTFE membrane, the TENG could yield a stabilized *V*_OC_ of 900 V, a short‐circuit current density (*J*_SC_) of 20 mA m^−2^, and a charge density of 149 µC m^−2^, at an impact force of 50 N and a contact frequency of 5 Hz with the separation distance of 5 mm.

### 2.2 FEP‐Based TENGs

**Figure 6:**
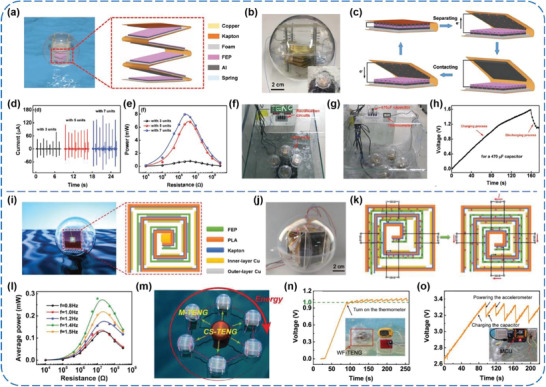
a–h) Spherical TENGs based on spring‐assisted multilayered structure for efficient water wave energy harvesting. Reproduced with permission.^[^ ^47^ ^]^ Copyright 2018, Wiley‐VCH. i–o) Whirling‐folded TENGs with high average power for water wave energy harvesting. Reproduced with permission.^[^ ^48^ ^]^ Copyright 2019, Wiley‐VCH.

Similarly, with all fluorine atoms in the main chain of molecules, FEP also owns considerably strong electrophilic capabilities and has been frequently used as an electronegative material in triboelectric pairs.^[^ ^47^, ^48^, ^49^, ^50^, ^51^ ^]^ Xiao et al. reported a type of spherical TENGs based on spring‐assisted multilayered structure to efficiently scavenge the low‐frequency, irregular, and “random” water wave energy.^[^ ^47^ ^]^ As presented in **Figure** **6**a, a zigzag multilayered TENG with five basic units is attached on the top side of a 4 mm acrylic block, and pressed by two 2 mm thick circular acrylic blocks with a copper block of 100 g in between. Triggered by water waves, periodic movements of the copper mass block caused continuous contact‐separation motions between the FEP film and Al foil (Figure 6c). At a water wave frequency of 1 Hz and an amplitude of 2.5 V, a TENG with seven units could yield outputs of 120 µA, 0.67 µC, 560 V, and 7.96 mW, which are much larger than those of previousy reported rolling spherical TENGs (Figure 6d,e).^[^ ^52^, ^53^, ^54^ ^]^ For potential applications, four such spherical TENGs were integrated into an array by linking with rigid strings to convert energy from water wave. Figure 6f shows that numerous LEDs with a “TENG” pattern could be lightened up by the array in a water tank. Also, the TENG array was used to charge a capacitor of 470 µF and further successfully powered an electronic thermometer (Figure 6g,h). In addition, An et al. developed a whirling‐folded TENG (WF‐TENG) by 3D printing and printed circuit board technologies.^[^ ^48^ ^]^ The flexible vortex structure of the WF‐TENG with two basic units in each face is clearly shown in Figure 6i. In order to bring about contact and separation between the FEP and Cu, a mass block was placed in the center of polylactic acid (PLA) skeleton, which could rock when the TENG was subjected to external triggering (Figure 6k). At a water wave of 10 cm in height and 1.4 Hz, the WF‐TENG with five basic units in each face was able to yield a maximum peak power of 6.5 mW and an average power of 0.28 mW (Figure 6l), which could charge capacitors to drive a digital thermometer for measuring the water temperature and to realize a self‐powered monitoring system for preventing the TENG network from possible damage in severe environments (Figure 6n,o). In addition, a self‐charge‐supplement WF‐TENG network was proposed, which consists of a charge‐supplement TENG (CS‐TENG) and several surrounding main TENGs (M‐TENGs), as shown in Figure 6m. Under normal working condition of the network, the CS‐TENG can continuously replenish charges for the M‐TENGs and it thus simultaneously improves the output performance and the stability of the network for possibly scavenging large‐scale blue energy.

**Figure 7:**
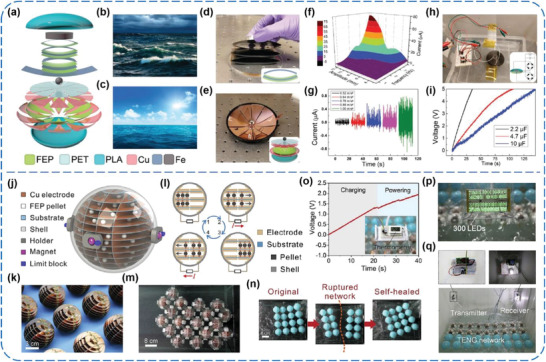
a–i) Oblate spheroidal TENGs for all‐weather blue energy harvesting. Reproduced with permission.^[^ ^49^ ^]^ Copyright 2019, Wiley‐VCH. j–q) Macroscopic self‐assembly network of encapsulated high‐performance TENGs for water wave energy harvesting. Reproduced with permission.^[^ ^50^ ^]^ Copyright 2019, Elsevier.

More particularly, Liu et al. designed a novel oblate spheroidal TENG (OS‐TENG) assembled by two triboelectric parts for harvesting blue energy from both situations of rough and tranquil seas (**Figure** **7**a).^[^ ^49^ ^]^ The upper part of the OS‐TENG is based on spring steel plates with FEP as one of triboelectric materials, which is able to produce considerable power in rough seas and occupy small space simultaneously (Figure 7b,d). Yet, the lower part consists of an iron shot and two copper‐coated polymer films (FEP and polyethylene terephthalate (PET)), which can capture small wave energy from tranquil seas (Figure 7c,e). The output performances of both parts were investigated with respect to various mechanical parameters including frequency, amplitude, and acceleration of a linear motor, and rotate speed and slant angle of a rotary motor (Figure 7f,g). A maximum *V*_OC_ of 281 V and an *I*_SC_ of 76 µA could be achieved by one upper part at an amplitude of 12.5 mm and a frequency of 4 Hz. For practical applications, the output electricity generated from water waves can be utilized to charge different commercial capacitors, in which case, a 4.7 µF capacitor was charged to 5 V within 2 min and 150 s were needed for a 10 µF capacitor (Figure 7h,i). Besides, the FEP pellets can be also used as a triboelectric material for mechanical energy conversion. Yang et al. developed a high‐performance TENG that possesses an 3D‐layered electrode structure with a certain number of intercalated FEP pellets, as schematically illustrated in Figure 7j–l.^[^ ^50^ ^]^ By a rationally designed self‐adaptive magnetic (SAM) joints, macroscopic networks made of this kind of TENGs demonstrated self‐assembly, self‐healing, and facile reconfiguration capabilities (Figure 7m,n). For a single TENG, average power densities of 8.69 and 2.05 W m^−3^ could be obtained under ideal agitations and in water, respectively, indicating excellent mechanical energy‐harvesting performance compared to existing similar devices.^[^ ^53^, ^55^, ^56^ ^]^ Three intuitive demonstrations of water wave energy harvesting by the TENG network were successfully carried out, as shown in Figure 7o–q, including lighting up 300 LEDs and powering a thermometer and a wireless transmitter. What is more, Zhu et al. reported a 2D‐structured triboelectric generator (TEG) for generating energy from rotary motions by using FEP and Cu as triboelectric materials.^[^ ^51^ ^]^ Enabled by two radial‐arrayed fine electrodes that are complementary on the same plane, the TEG can effectively harvest various ambient mechanical motions, including normal body movement, light wind, and water flow. At a rotation speed of 3000 r min^−1^, a TEG with a diameter of 10 cm could yield a *V*_OC_ of 850 V and an *I*_SC_ of 3 mA, as well as an average output power density of 19 mW cm^−2^ under a 0.8 MΩ load. By integrating the TEG with a power management circuit, a completely constant direct‐current source (≈5 V) was built as power supplies for various commercial electronics, such as spot lights, wireless emitters, multifunction digital clocks, and even cellphones.

### 2.3 PVDF‐Based TENGs

**Figure 8:**
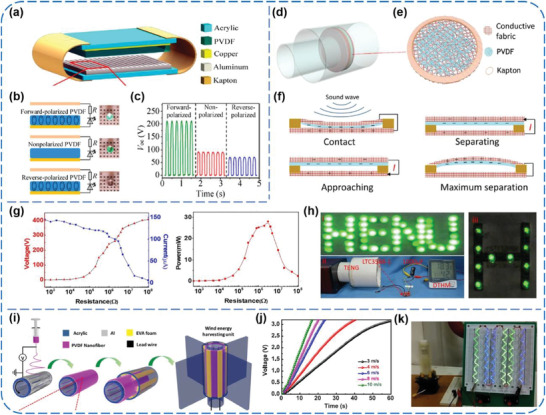
a–c) Vertical contact‐separation‐mode TENGs using pristine PVDF film as triboelectrification layer. Reproduced with permission.^[^ ^59^ ^]^ Copyright 2014, Tsinghua University Press and Springer‐Verlag GmbH Germany, part of Springer Nature. d–h) Electrospun PVDF nanofibers‐based TENGs for acoustic energy harvesting. Reproduced with permission.^[^ ^60^ ^]^ Copyright 2019, Elsevier. i–k) A coaxial rotatory freestanding TENG with an annular electrospinning PVDF nanofibrous membrane and rotatable Al electrodes as triboelectric materials for wind energy harvesting. Reproduced with permission.^[^ ^61^ ^]^ Copyright 2018, Elsevier.

PVDF and its copolymers as a kind of highly non‐reactive PMs that possess superior piezoelectric properties, low acoustic impedance, adequate mechanical robustness, and desirable flexibility have become one of the most popular PMs both in piezoelectric and triboelectric energy‐harvesting technologies.^[^ ^57^, ^58^, ^59^, ^60^, ^61^, ^62^, ^63^, ^64^, ^65^ ^]^ Bai et al., for the first time, used the PVDF pristine film without any modifications as a triboelectric layer to construct vertical contact‐separation mode TENGs, as shown in **Figure** **8**a.^[^ ^59^ ^]^ The effects of different poling processes on output performance of the TENGs were investigated (Figure 8b). The *V*_OC_ could be either enhanced to 240% or reduced to 70% depending on the direction of dipole moments inside the poled PVDF film (Figure 8c), which reveals that triboelectric charge transfer upon contact‐electrification might be subject to the modification of tribosurface potential. Since then, a few attempts have been undertaken to further enhance the outputs of the PVDF‐based TENGs by adding various active dielectric and piezoelectric nanomaterials into PVDF film, such as ZnSnO_3_ nanocubes, BaTiO_3_ nanoparticles (NPs), and silver nanowires (NWs), among others.^[^ ^66^, ^67^, ^68^ ^]^ In addition, for enlarging the friction area during contact, PVDF nanofibers‐based membranes have always been prepared through electrospinning and used as triboelectric layers.^[^ ^60^, ^61^, ^62^, ^63^, ^64^ ^]^ Chen et al. reported a novel contact‐separation‐mode TENG based on electrospun PVDF nanofibers and conductive fabrics, and integrated it with polyvinyl chloride (PVC) polymer tubes, which could harness acoustic energy with the frequency from 20 to 1000 Hz in ambient environment (Figure 8d–f).^[^ ^60^ ^]^ Under the sound waves of 170 Hz and 115 dB, the TENG could generate a *V*_OC_ of 400 V and an *I*_SC_ of 175 µA, with a maximum peak power density of 7 W m^−2^ (Figure 8g). To demonstrate practical applicability of the TENG, it was fixed on a small mechanical pump. When the mechanical pump is operating, the power generated from the pump noise is enough to light 10 LEDs and drive a digital temperature‐humidity meter (Figure 8h), showing great potential for the TENG using in real environments. Also, Ren et al. presented a wind energy harvester on the basis of a coaxial rotatory freestanding TENG (CRF‐TENG). The TENG adopted an annular electrospinning PVDF nanofibrous membrane and rotatable Al electrodes as triboelectric materials (Figure 8i).^[^ ^61^ ^]^ By using the air flow generated from an air gun to blow the wind blades of the TENG, its electric output was investigated in the light of wind velocity. The *I*_SC_ value substantially increased with increasing the wind velocity, and reached about 40 µA at a wind velocity of 10 m s^−1^. As well, the generated electricity was used to charge capacitors and drive LED bulbs, as shown in Figure 8j,k. More importantly, the converted power from the wind by the TENG could be also used to split water for H_2_ production, in which a H_2_ generation rate of 6.9685 µL min^−1^ was achieved in 1 M KOH solution at a wind velocity of 10 m s^−1^. This kind of fully self‐powered water splitting system pioneers a new efficient and scalable way to convert mechanical energy into hydrogen energy.

### 2.4 Polydimethylsiloxane (PDMS) and Other Silicones‐Based TENGs

**Figure 9:**
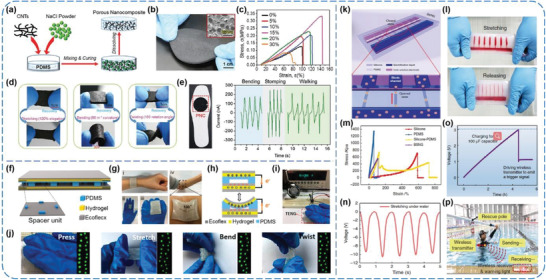
a–e) Synthesis process and property characterization of a stretchable porous nanocomposite. Reproduced with permission.^[^ ^69^ ^]^ Copyright 2017, Wiley‐VCH. f–j) Toughly bonded elastomer/hydrogel hybrids‐based TENGs. Reproduced with permission.^[^ ^70^ ^]^ Copyright 2018, American Chemical Society. k–p) A bionic stretchable nanogenerator for underwater sensing and energy harvesting. Reproduced with permission.^[^ ^71^ ^]^ Copyright 2019, Springer Nature.

By right of superior flexibility and stretchability, adequate mechanical robustness, easy and scalable fabrication, desirable processability, and low cost, (PDMS) as well as other silicones has already been one of the most attractive PMs and extensively used in many fields, such as flexible/stretchable electronic/optical systems, portable/wearable/implantable devices, soft lithography, encapsulation, and hydrophobic coatings. In recent years, for triboelectric energy‐harvesting technology, PDMS, and several silicones have often been adopted as friction materials for constructing varieties of conventional or stretchable TENGs.^[^ ^54^, ^69^, ^70^, ^71^, ^72^, ^73^, ^74^, ^75^, ^76^, ^77^, ^78^ ^]^ Fan et al. reported a stretchable porous nanocomposite (PNC) for generating energy from various mechanical stimuli, which is made of a multiwalled carbon nanotubes (CNTs) network and a PDMS matrix.^[^ ^69^ ^]^ Synthesized by a facile two‐step sacrificial template method, the PNC is densely packed with plenty of cavities that are equivalent to micro‐scaled TENG units (**Figure** **9**a,b). As given in Figure 9c, the PNC can be stretched to an elongation of 143%. Under compression at a force of 60 N, the PNC (2.5 × 2.5 × 0.5 cm^3^) with the CNTs content of 15 wt% and the porosity of 70% could bring in a *V*_OC_ of 60 V and an *I*_SC_ of 180 nA. Besides, it is also capable of harvesting other mechanical interactions, including stretching, bending, and twisting (Figure 9d). For potential in harvesting human body motions, a round PNC was taped onto a shoe insole to scavenge energy from human walking (Figure 9e), during which the generated electricity was able to directly lighten a few dozen commercial LEDs. Liu et al. developed a soft and stretchable TENG made of toughly bonded elastomer/hydrogel hybrids.^[^ ^70^ ^]^ In the case of two‐electrode‐mode TENG (Figure 9f), a polyacrylamide−sodium alginate (PAAm−alginate) hydrogel film sealed inside elastomer layer with interfacial modification of benzophenone treatment was adopted as both top and bottom electrodes. And the middle Ecoflex and PDMS films serve as tribo‐positive and tribo‐negative layers, respectively. Enabled by these soft materials, the TENG owns superior flexibility, which makes it able to be conformally attach to diverse curvy surfaces of human body (Figure 9g). Under pressing, stretching, bending, or twisting motions, the hydrogel‐elastomer‐based TENG could simultaneously lighten 20 green LEDs connected in series (Figure 9j). Likewise, the electrical energy produced from the TENG could be charged and stored in capacitors to power up portable electronics, such as an electronic watch (Figure 9i), explicitly showing possibility for the TENG to be integrated with deformable, portable, and wearable electronic devices. More recently, Zou et al. developed an electric eel‐inspired bionic stretchable nanogenerator (BSNG) for underwater energy harvesting.^[^ ^71^ ^]^ This kind of TENG mimics the ion channel structure in electrocyte cytomembrane of an electric eel.^[^ ^71^ ^]^ As schematically shown in Figure 9k, the BSNG comprises a PDMS‐silicone‐based electrification top layer that contains a series of controllable channels and an electrification liquid‐filled (deionized water) chamber, and an induction bottom layer made of two ionic solution (NaCl) electrodes. Under stretching and releasing operations, the multiple channels can be opened and closed correspondingly with the electrification liquid flowing into the chambers and flowing back into the reservoir (Figure 9l). Based on electrostatic induction between two ionic solution electrodes, the BSNG, thus, can generate alternating current sustainably, which is different from previously reported generators harnessing ion‐concentration gradients.^[^ ^79^, ^80^, ^81^ ^]^ At a tensile frequency of 1 Hz and a tensile strain of 50%, the *V*_OC_, *I*_SC_, and *Q*_SC_ from the BSNG under water could reach 10 V, 36.5 nA, and 2 nC, respectively (Figure 9n). For possible underwater applications, an undersea rescue system was built by the BSNGs, a wireless transceiver module, and a warning light. A 100 µF capacitor was charged from 0 to 3 V by four rectified BSNGs worn on human body in about 4 h, which could be used to drive the wireless transmitter to emit a trigger signal one time and to remotely switch the red warning light (Figure 9o,p). This work provides a novel TENG structure that takes advantage of PDMS‐silicone‐based elastomer to achieve stretching motion‐caused contact‐electrification for harvesting biomechanical energy from human body, and demonstrates its great potential in various possible underwater‐sensing applications.

**Figure 10:**
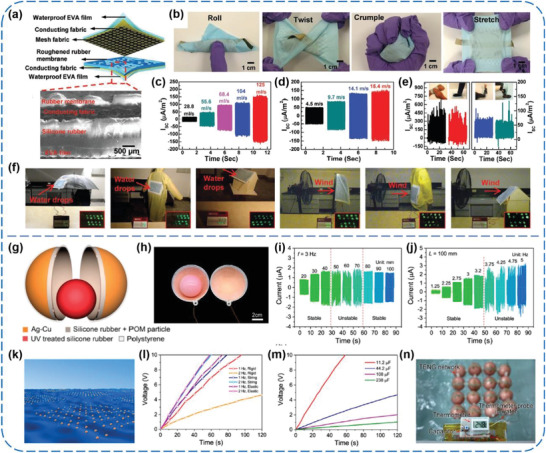
a–f) Waterproof‐fabric‐based multifunctional TENGs for universally harvesting energy from raindrops, wind, and human motions. Reproduced with permission.^[^ ^72^ ^]^ Copyright 2019, Wiley‐VCH. g–n) Coupled silicone rubber‐based TENG networks for efficient water wave energy harvesting. Reproduced with permission.^[^ ^54^ ^]^ Copyright 2018, American Chemical Society.

Besides, Lai et al. presented a waterproof‐fabric‐based multifunctional TENG (WPF‐MTENG) that operates between a roughened silicone rubber membrane and a mesh fabric, as illustrated in **Figure** **10**a.^[^ ^72^ ^]^ The soft and elastic features of ethylene‐vinyl acetate substrates, conductive fabrics, and silicone rubbers not only empower the WPF‐MTENG to be readily crumpled, twisted, rolled up, and stretched, but also make it capable of turning various gentle interactions including human motions, winds, and even raindrops into electric outputs (Figure 10b–e). In order to prove its practical applicability, the WPF‐MTENG was employed on the appearance of an umbrella, a raincoat, a roof, and a flag to collect rainfall and wind energy, respectively. As shown in Figure 10f, tens of LEDs could be lighted up by the harvested electric energy, intuitively manifesting the promise of the WPF‐MTENGs as alternative energy collectors toward wearable technologies. Moreover, Xu et al. used both silicone rubbers as triboelectric materials to develop a ball‐shell structured TENG for scavenging water wave energy, in which a UV‐treated silicone ball acts as tribo‐positive one and a polyformaldehyde‐doped silicone layer serves as tribo‐negative one (Figure 10g,h).^[^ ^54^ ^]^ The effects of displacement amplitude, frequency, and orientation of external harmonic agitations on the outputs of a single TENG were studied, as given in Figure 10i,j. And three kinds of linkage strategies for the TENG networks with 4 × 4 arrays were compared, which revealed that flexible connections among each TENG unit were better than the rigid one (Figure 10l,m). For application demonstration, a string connected network was successfully utilized to charge a 108 µF capacitor and further to run a thermometer, as presented in Figure 10n.

### 2.5 Polyimide (PI)‐Based TENGs

**Figure 11:**
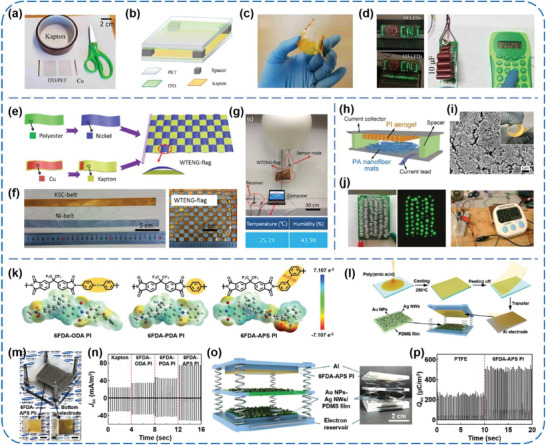
a–d) Facile and robust TENGs assembled using off‐the‐shelf materials. Reproduced with permission.^[^ ^82^ ^]^ Copyright 2019, Elsevier. e–g) Freestanding flag‐type TENGs for harvesting high‐altitude wind energy from arbitrary directions. Reproduced with permission.^[^ ^83^ ^]^ Copyright 2016, American Chemical Society. h–j) PI aerogel film‐based TENGs. Reproduced with permission.^[^ ^85^ ^]^ Copyright 2018, American Chemical Society. k–p) Dual inductive and resonance effects‐controlled highly transparent PI‐based high‐output TENGs. Reproduced with permission.^[^ ^87^ ^]^ Copyright 2019, Wiley‐VCH.

PI is a type of widely used PM in the fields of packaging, coating, thermal insulation, adhesive agents, and liquid crystal devices, among others. Due to the existing fluorine atoms and acyl groups in its molecules, PI has always been adopted as an electron‐withdrawing material for TENGs.^[^ ^82^, ^83^, ^84^, ^85^, ^86^, ^87^, ^88^ ^]^ Mallineni et al. prepared a low‐cost (≈$0.06 cm^−2^), ultra‐simple, easy‐scalable, and robust (>20 000 cycles) TENG using off‐the‐shelf materials, including commercial PET/ITO and Kapton films (**Figure** **11**a–c).^[^ ^82^ ^]^ By facilely inserting and then removing a piece of cellulose paper in between the PET/ITO and Kapton, enhanced outputs of 480 V and 1.7 mW were acquired from the TENG under a vertical pushing force of 50 N at 2 Hz, which were competent to light up red/green LEDs, charge capacitors, or intermittently drive an eight‐digit handheld calculator (Figure 11d). Zhao et al. developed a novel woven TENG flag (WTENG‐flag), which is able to generate power from wind in arbitrary directions (Figure 11e,f).^[^ ^83^ ^]^ The WTENG‐flag works based on contact‐electrification between Ni‐coated polyester fabrics (Ni belts) and Kapton film‐sandwiched Cu belts (KSC belts), and its outputs can easily be enhanced by stacking such 2D layer structures in parallel connections. For potential applications, the flag was successfully utilized to power a sensing node of a high‐altitude platform with temperature/humidity sensing/telecommunicating capabilities, as shown in Figure 11g. Furthermore, Mi et al. developed a new type of high‐performance TENG that made of a PI aerogel film and highly porous nylon nanofiber mats (Figure 11h,i).^[^ ^85^ ^]^ The electric outputs of the TENG were optimized in terms of the thickness of triboelectric materials, and relatively high outputs of 115 V and 9.5 µA were obtained from the TENG (≈2 cm^2^ in active area) in response to a small pressure (≈30 kPa). In this case, a peak power density of 1.84 W m^−2^ was obtained at an external load of 4.7 MΩ. Also, the TENG was capable of easily lighting 60 LEDs and rapidly charging capacitors to drive a timer (Figure 11j).

More recently, Lee et al. synthesized three kinds of PI‐based polymers (6FDA‐ODA, 6FDA‐PDA, and 6FDA‐APS PI) by introducing electron‐withdrawing or electron‐donating functional groups into the backbone of PI to fabricate high‐output TENGs (Figure 11k).^[^ ^87^ ^]^ Through attaching the PI‐based films to an Al electrode and then integrating them with an Au NPs‐decorated PDMS/Ag NWs composite layer, respectively, several contact–separation‐mode TENGs were constructed, as shown in Figure 11l,m. The results of density functional theory calculations indicated that the 6FDA‐APS PI possessed the most negative electrostatic potential and the low‐lying lowest unoccupied molecular orbital level, which could render the best charges retention characteristics and enhanced charge transfer capability, respectively (Figure 11k,n). Therefore, the generated charge density observably increased by about seven times compared to that of the commercial PI film‐based TENG. In three‐layered configuration (Figure 11o), a maximum charge density of about 512 µC m^−2^ could be obtained under a compression force of 30 N and 3 Hz (Figure 11p). To the best of our knowledge, this value is the highest charge density of the TENGs in practical environments without ions injection process and additional circuitry. Additionally, the charge density was increased to about 860 µC m^−2^ with assistance of an ion's injection process under the same mechanical agitations. This work brings a deeper insight into the charge retention characteristic and charge transfer capability of PI‐based triboelectric materials, and inspires us to better improve the TENGs’ performance from the material design and modification point of view.

### 2.6 PET‐Based TENGs

**Figure 12:**
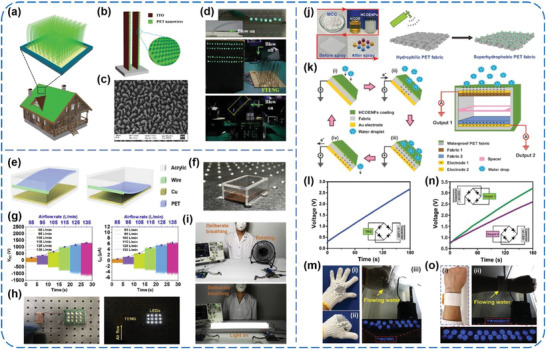
a–d) Lawn structured TENGs for scavenging sweeping wind energy on rooftops. Reproduced with permission.^[^ ^89^ ^]^ Copyright 2016, Wiley‐VCH. e–i) A breath‐driven TENG for human‐machine interaction. Reproduced with permission.^[^ ^90^ ^]^ Copyright 2019, Elsevier. j–o) Wearable all‐fabric‐based TENGs for water energy harvesting. Reproduced with permission.^[^ ^91^ ^]^ Copyright 2017, Wiley‐VCH.

Despite the weaker electron affinity compared to the abovementioned PMs that contain fluorine atoms, PET still has been adopted as one kind of tribo‐negative material owing to its decent flexibility and strength, good machinability, and low cost.^[^ ^89^, ^90^, ^91^, ^92^, ^93^, ^94^, ^95^, ^96^ ^]^ Zhang et al. reported lawn‐structured TENGs for harnessing wind energy on rooftops.^[^ ^89^ ^]^ As sketched in **Figure** **12**a, the TENGs array owns a kelp‐forest‐like morphology with numerous basic TENG units, that is, a pair of strips, each of which consists of an ITO‐coated PET film with an end staying freestanding and another end anchored onto the substrate (Figure 12b). Additionally, to increase the effective contact area, NW structures were prepared on the exposed PET surface via reactive ion etching (Figure 12c). Under an airflow at 27 m s^−1^, a TENG unit (10 × 2 cm^2^) could deliver a *V*_OC_ of 98 V, an *I*_SC_ of 16.3 µA, and a power density of 2.76 W m^−2^. For practical applications, the power converted from wind by a TENG array with 60 basic units was used to illuminate LEDs and a display board (Figure 12d), which explicitly proved the possibility of the TENGs using as a sustainable power source for some home‐use electronics and presented a solid step towards self‐powered home technology. Recently, Zhang et al. developed a breath‐driven, single‐electrode TENG made of a NW‐structured PET thin film and a Cu foil, as shown in Figure 12e,f.^[^ ^90^ ^]^ When air flows through the acrylic tube, the vibration could induce periodic contact–separation interactions between the PET thin film and Cu foil. The *V*_OC_ and *I*_SC_ both increased with increasing the airflow rate (Figure 12g). Under the airflow at 115 L min^−1^, the generated electric power could directly light up 12 LEDs, as exhibited in Figure 12h. Moreover, a smart, wireless, breath‐driven human–machine interface (HMI) system was constructed by integrating a TENG‐based self‐powered breathing sensor with signal processing and transmitting circuits (Figure 12i). This system is capable of transforming a deliberately strengthened breathing behavior to a control of electrical appliances, which makes the HMI interaction more captivating and convenient, especially for some disable people. What is more, Xiong et al. reported a wearable, self‐cleaning, and antifouling all‐fabric‐based TEG for harvesting water energy.^[^ ^91^ ^]^ As schematically illustrated in Figure 12j, the authors prepared hydrophobic cellulose oleoyl ester nanoparticles from microcrystalline cellulose as low‐cost and nontoxic coating materials to realize superhydrophobic PET fabrics. On this basis, two kinds of TEGs, that is, a water‐TEG (WTEG) and a packaged dual‐mode TEG (DMTEG), were prepared to collect water energy (Figure 12k). Under the impact from a tap water flow with controlled flowing rate, impact distance, and angle, the output performance of the WTEG and DMTEG was investigated. More significantly, the WTEG and DMTEG can be woven into a cotton glove and constructed as a wearable wristband, respectively, and further used to drive commercial LEDs through converting the water energy (Figure 12l–o). This work gives an attractive concept to achieve a fabric‐based TEG for water energy harvesting and qualifies it to be a promising power supply for various wearable systems.

### 2.7 Other Well‐Investigated Tribo‐Negative PMs in TENGs

**Figure 13:**
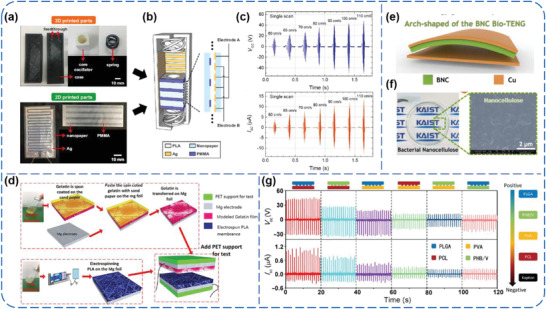
a–c) AP‐TENGs by using PMMA as tribo‐negative material. Reproduced with permission.^[^ ^97^ ^]^ Copyright 2018, Elsevier. d) Fully biodegradable TENGs based on nanostructured gelatin film and electrospun PLA. Reproduced with permission.^[^ ^98^ ^]^ Copyright 2018, Elsevier. e,f) Bio‐TENGs with BNC as tribo‐negative material. Reproduced with permission.^[^ ^99^ ^]^ Copyright 2017, Elsevier. g) Biodegradable TENGs based on four biodegradable polymers. Reproduced with permission.^[^ ^101^ ^]^ Copyright 2016, The American Association for the Advancement of Science.

In addition to the six commonly used electronegative PMs mentioned above, there are also other tribo‐negative PMs that have been well‐investigated for TENGs. Seol et al. reported an all‐printed TENG (AP‐TENG), which consists of a 3D‐printed PLA frame and two 2D‐printed plane layers (**Figure** **13**a).^[^ ^97^ ^]^ The tribo‐negative and positive materials are grating‐patterned poly(methyl methacrylate) (PMMA) and Ag films, respectively, and both of them use nanocellulose film as substrate. The AP‐TENG could convert external vibration into linear sliding contact between the PMMA and Ag (Figure 13b), and a maximum instantaneous voltage of 98.2 V and a maximum instantaneous current of 13.7 µA could be produced under a threshold vibration amplitude of 1 mm with an optimum frequency range of 30–60 Hz (Figure 13c). Furthermore, Pan et al. developed a fully biodegradable and high‐power density TENG by using a nanostructured gelatin film as tribo‐negative layer and an electrospun PLA nanofiber membrane as tribo‐positive layer, as shown in Figure 13d.^[^ ^98^ ^]^ Under a contact force of 50 N and 5 Hz with a separation distance of 4 mm, the *V*_OC_ and *I*_SC_ density could reach up to 500 V and 10.6 mA m^−2^, respectively, and a maximum power density of 5 W m^−2^ was achieved for the TENG. Including the transitional metal (Mg) electrodes, all of the materials in the TENG could be completely degraded into water after 40 days, making the TENG a promising green micro‐power source for self‐powered systems. Noteworthily, as for some PMs that possess a relatively weak electron affinity, such as cellulose and polyvinyl alcohol (PVA), they can be used either as tribo‐negative or as tribo‐positive materials, which depend on their counterparts.^[^ ^99^, ^100^, ^101^ ^]^ Kim et al. reported a bio‐TENG based on eco‐friendly and naturally abundant bacterial nanocellulose (BNC), as depicted in Figure 13e.^[^ ^99^ ^]^ Through a solubilization process, a biocompatible, transparent, and flexible BNC film was prepared and used as tribo‐negative part in the bio‐TENG (Figure 13f). Under a light touch of 16.8 N, accumulative charge amount of 8.1 µC m^−2^ and a peak power density of 4.8 mW m^−2^ could be obtained at a load of 1 MΩ, suggesting the biomaterial, BNC, is capable of serving as triboelectric materials to convert possible mechanical energies. In addition, Zheng et al. adopted four biodegradable PMs, including poly(L‐lactide‐*co*‐glycolide) (PLGA), poly(3‐hydroxybutyric acid‐*co*‐3‐hydroxyvaleric acid) (PHB/V), poly(caprolactone) (PCL), and PVA, to fabricate biodegradable TENGs.^[^ ^101^ ^]^ By using a PI film as reference contact layer, the relative ability to gain electrons was ranked as PCL > PVA > PHB/V > PLGA, indicating that PCL and PVA are more likely to act as tribo‐negative one among them (Figure 13g).

### 2.8 Nylon‐Based TENGs

**Figure 14:**
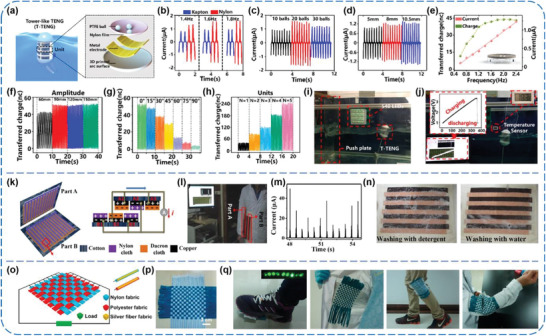
a–j) High power density tower‐like TENGs for harvesting arbitrary directional water wave energy. Reproduced with permission.^[^ ^45^ ^]^ Copyright 2019, American Chemical Society. k–n) Wearable TENGs for powering the portable electronic devices. Reproduced with permission.^[^ ^103^ ^]^ Copyright 2015, American Chemical Society. o–q) Woven structured TENGs for harvesting biomechanical energy. Reproduced with permission.^[^ ^104^ ^]^ Copyright 2014, American Chemical Society.

Nylon is one of the most common fabric PMs that possesses excellent mechanical robustness, softness, and fabricability. For triboelectric energy harvesting, nylon always acts as the tribo‐positive material by virtue of its fairly strong electron‐donating ability, even compared with metals.^[^ ^19^, ^45^, ^46^, ^102^, ^103^, ^104^, ^105^, ^106^, ^107^ ^]^ Zhang and co‐workers proposed a high‐power density tower‐like TENG (T‐TENG) by utilizing nylon film and PTFE balls as triboelectric materials for harnessing arbitrary directional water wave energy.^[^ ^45^ ^]^ As shown in **Figure** **14**a, the T‐TENG is made of a tubular acrylic block with the shell diameter of 100 mm and internal multiple TENG units, each of which comprises a few PTFE balls, a melt adhesive reticulation nylon film, two Al electrodes, as well as a 3D printed arc shell. By using a linear motor, the effects of a series of factors on output performance of the T‐TENG were comprehensively measured, including dielectric materials, the number and diameter of PTFE balls, the curvature of the 3D printed arc shell, the frequency, amplitude, and direction of external agitations, and the number of TENG units (Figure 14b–h). Based on optimized structural parameters, the power density of the T‐TENG can increase linearly from 1.03 to 10.6 W m^−3^ by increasing the TENG units connected in parallel from 1 to 10 without rectifiers in one block, exhibiting a relatively high space utilization compared to several previous works.^[^ ^108^, ^109^ ^]^ For demonstrations of harvesting water wave energy, a T‐TENG with five units was pulled by a rope to the bottom of a water tank. As shown in Figure 14i,j, the produced electric energy could simultaneously lighten more than 500 LEDs and also could charge a 100 µF capacitor to power a thermometer. Similarly, nylon fabrics have been adopted as triboelectric materials due to the merits of flexible, wearable, and washable characters. Cui et al. developed a cloth‐based wearable TENG that made of cotton cloth substrates, nylon, and Dacron fabrics for scavenging biomechanical energy (Figure 14k).^[^ ^103^ ^]^ Both the fabrics were naturally knitted with numerous threadlike fibers, rendering abundant microstructures to boost the outputs of the device. By properly attaching two parts of the TENG on the inner forearm and waist, respectively, the TENG was capable of generating electric energy from the motions of arm swinging with a rectified outputs of 0.7 V and 50 µA (Figure 14l,m). Importantly, there was no obvious damage to the outputs, structure, and appearance of the TENG after washing (Figure 14n), indicating great potential for using in wearable electronic systems. Additionally, Zhou et al. fabricated a woven‐structured TENG (W‐TENG) by using three kinds of fabrics, including commodity nylon, polyester, and conductive silver fiber fabric (Figure 14o,p).^[^ ^104^ ^]^ Featuring advantages of decent flexibility, breathability, wearability, and washability, the W‐TENG could not only be conformally attached on clothes but also move freely without any constraint, making it suitable for wearable electronic systems. For practical applications, the W‐TENG was successively sewn on shoes, coats, and trousers to convert biomechanical energy from various human motions, such as stepping, shaking, and bending of joints (Figure 14q).

**Figure 15:**
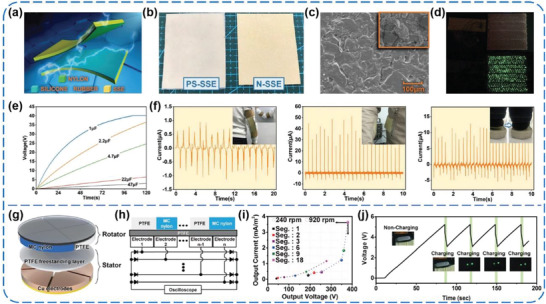
a–f) A nonmetallic stretchable nylon‐modified high performance TENG for energy harvesting. Reproduced with permission.^[^ ^105^ ^]^ Copyright 2019, Wiley‐VCH. g–j) A multi‐phase TENG with sustainable DC outputs. Reproduced with permission.^[^ ^107^ ^]^ Copyright 2018, Royal Society of Chemistry.

In addition to common and fabric films, nylon powders were also used as triboelectric materials. Qian et al. presented a nonmetallic stretchable TENG based on conductive glass microspheres‐doped stretchable silicone electrodes (SSE), which were covered with nylon powders (N‐SSE) and pure silicone rubber (PS‐SSE), respectively (**Figure** **15**a–c).^[^ ^105^ ^]^ Under periodic contact‐separation motions at a force of 100 N and 3 Hz, the *V*_OC_ and *I*_SC_ of the nylon‐modified TENG (NM‐TENG) could reach 1.17 kV and 138 µA, respectively. At the same time, a maximum instantaneous power density of 11.2 W m^−2^ could be obtained at a matched load of 10 MΩ. The outputs after rectification were capable of directly lighting 480 LEDs and driving an electronic clock parallel with a capacitor (Figure 15d,e). Furthermore, by attaching the NM‐TENG on different positions of human body, such as elbow, waist, and heel, it could convert various body motions into considerable electric outputs, providing a good potential in wearable energy‐harvesting systems (Figure 15f). Particularly, for the first time, Ryu et al. introduced a multi‐phase TENG (MP‐TENG) that yields almost constant direct current (DC) with a crest factor as low as 1.26.^[^ ^107^ ^]^ The TENG is designed on the basis of a conventional rotation type structure, which comprises an alternating component of PTFE and mono‐cast nylon as the rotator and a whole PTFE with multiple back electrodes as the stator (Figure 15g). By pre‐rectifying and superimposing the outputs from all electrodes, a five‐phase MP‐TENG in a configuration of 18 segments could produce outputs of 3.6 mA m^−2^, 380 V, and 4.9 W m^−2^ (Figure 15h,i). In this case, the MP‐TENG was successfully used to power a wearable electronic device through a commercial power management module (Figure 15j), and to operate a temperature sensor, as well as to charge a Li‐ion battery. The results indicate that this type of TENG made of nylon and PTFE films is promising for directly powering a variety of commercial electronics and possible self‐powered systems.

### 2.9 Cellulose‐Based TENGs

**Figure 16:**
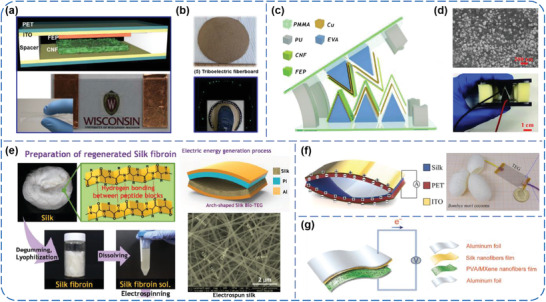
a,b) TENGs and power‐boards from CNF and recycled materials. Reproduced with permission.^[^ ^110^ ^]^ Copyright 2016, Elsevier. c,d) Chemically functionalized CNF‐based gear‐like TENGs. Reproduced with permission.^[^ ^111^ ^]^ Copyright 2019, Elsevier. e) Silk nanofiber‐networked bio‐TEGs. Reproduced with permission.^[^ ^114^ ^]^ Copyright 2016, Wiley‐VCH. f) Silk‐fibroin‐based transparent TENGs. Reproduced with permission.^[^ ^115^ ^]^ Copyright 2016, Elsevier. g) All‐electrospun flexible TENGs based on silk fibroin and PVA nanofiber films. Reproduced with permission.^[^ ^116^ ^]^ Copyright 2019, Elsevier.

Cellulose is one of the most abundant natural PMs with low cost, light weight, high strength, biodegradability, and good chemical modification property, which also exhibits a high tendency of losing electrons due to its inclusion of plentiful oxygen atoms.^[^ ^110^ ^]^ Thus, cellulose‐based materials have become good candidates for tribo‐positive part of the TENGs.^[^ ^110^, ^111^, ^112^, ^113^ ^]^ Yao et al. prepared a transparent and flexible cellulose nanofibrils (CNF) film with nanoscale surface roughness via a vacuum filtration method, and paired it with a FEP film to assemble TENG device, as schematically illustrated in **Figure** **16**a.^[^ ^110^ ^]^ The average peak *V*_OC_ and *I*_SC_ of the CNF‐based TENG could reach up to 5 V and 7 µA under a constant force, respectively. The TENG was further integrated with recycled cardboard fibers to fabricate a triboelectric fiberboard (Figure 16b), which could produce electric outputs of 30 V and 90 µA when subjected to a normal human step. Besides, Zhang et al. designed a gear‐like TENG using a polyethyleneimine‐modified and Ag NPs‐coated CNF film as the tribo‐positive layer.^[^ ^111^ ^]^ As shown in Figure 16c, the entire frame of the TENG was made of PMMA plates and polyurethane (PU) sponge as spacers. By virtue of enhanced electron‐releasing character from amino groups and nanoscale surface morphology from Ag NPs (Figure 16d), the TENG with three pairs could generated a maximum *V*_OC_ of 286 V and a maximum *I*_SC_ of 4 µA under a contact force of 10 N and 2 Hz, capable of directly lighting up 60 LEDs. Also, Roy et al. developed a high‐performance cellulosic TENG through the modification of allicin grafting onto CNFs surface.^[^ ^112^ ^]^ With the presence of high dipolar sulfoxide groups, the CNF film turned to be highly tribo‐positive, and thus high outputs of 7.9 V and 5.13 µA were achieved when coupled with a tribo‐negative PVDF layer, which were ≈6.5 times higher than those of the pristine CNF‐based TENG.

### 2.10 Silk‐Based TENGs

Silk, one of naturally abundant and eco‐friendly biomaterials, is a kind of natural protein fibers with several distinctive properties, including good biocompatibility, mechanically high strength and robustness, and decent electric insulativity.^[^ ^114^ ^]^ In recent years, silk has been gradually applied to construct TENGs and proved to be a superior trio‐positive material.^[^ ^114^, ^115^, ^116^ ^]^ Kim et al. for the first time adopted an electrospun film of silk fibroin nanofibers to build a bio‐TEG (Figure 16e).^[^ ^114^ ^]^ The electric outputs of 1.86 µC m^−2^ and 4.3 mW m^−2^ were obtained at a load resistance of 5 MΩ from the bio‐TEG. Further, owing to unique molecular structure of the fibroin and fracture‐tolerant behavior of the nanofiber networks, the bio‐TEG simultaneously exhibited extraordinary mechanical durability and reliability even over 25 000 test cycles. Zhang et al. constituted a triboelectric pair by using a silk fibroin film on one side, and a PET substrate on the other side.^[^ ^115^ ^]^ As shown in Figure 16f, this kind of flexible, light weight, transparent, and stable TEG could produce maximum outputs of 268 V, 5.78 µA, and 193.6 µW cm^−2^ onto a matched load of 40 MΩ under finger pressing, which were sufficient to independently operate two LCDs and actuate a micro‐cantilever. Additionally, Jiang et al. fabricated a flexible and robust TENG with all‐electrospun triboelectric layers, that is, a film of silk fibroin nanofibers as the tribo‐positive material and a MXene nanosheets‐modified PVA film as the tribo‐negative material (Figure 16g).^[^ ^116^ ^]^ Under a vertical force of 10 N and 10 Hz, the TENG could render an instantaneously maximum peak power density of 108.76 µW cm^−2^ with a load resistance of 5 MΩ, and it was further successfully used to power an electrowetting on dielectric chip for actuating droplet transport.

### 2.11 Other Well‐Investigated Tribo‐Positive PMs in TENGs

**Figure 17:**
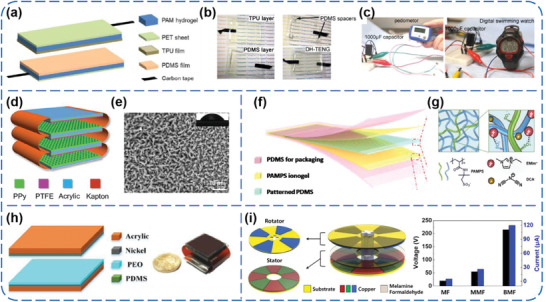
a–c) Flexible semitransparent dual‐electrode hydrogel‐based TENGs. Reproduced with permission.^[^ ^117^ ^]^ Copyright 2020, Royal Society of Chemistry. d,e) All‐plastic‐material‐based TENGs using a micro/nanostructured Ppy as tribo‐positive layer. Reproduced with permission.^[^ ^119^ ^]^ Copyright 2016, Wiley‐VCH. f,g) Ionogel‐based transparent and stretchable TENGs. Reproduced with permission.^[^ ^120^ ^]^ Copyright 2019, Elsevier. h) PEO as a new tribo‐positive material for high power TENGs. Reproduced with permission.^[^ ^121^ ^]^ Copyright 2018, Elsevier. i) Butylated MF as a durable and highly tribo‐positive material for high output TENGs. Reproduced with permission.^[^ ^123^ ^]^ Copyright 2019, Royal Society of Chemistry.

Apart from abovementioned cases, there are still many tribo‐positive PMs that have been used for the fabrication of TENGs, such as thermoplastic polyurethane (TPU),^[^ ^117^, ^118^ ^]^ polypyrrole (PPy),^[^ ^119^ ^]^ ionic gel,^[^ ^120^ ^]^ polyethylene oxide (PEO),^[^ ^121^, ^122^ ^]^ melamine formaldehyde (MF),^[^ ^123^ ^]^ PVA,^[^ ^124^ ^]^ peptides,^[^ ^125^ ^]^ and so on. Jing et al. reported a semitransparent dual‐electrode hydrogel‐based TENG, with a TPU film as tribo‐positive layer, a PDMS film as tribo‐negative layer, NaCl‐containing polyacrylamide (PAM) hydrogel films as electrodes, and PET sheets as substrates (**Figure** **17**a–c).^[^ ^117^ ^]^ High outputs of 311.5 V and 32.4 µA accompanied with a maximum power density of 2.7 W m^−2^ were achieved, which enabled the TENG to charge capacitors quickly for powering small electronics. Wang et al. used a micro/nanostructured PPy film with a hollow horn‐like surface morphology as tribo‐positive layer to build an all‐plastic‐materials‐based TENG (Figure 17d,e).^[^ ^119^ ^]^ With each active area of 1.7 × 1.7 cm^2^, a maximum output power of 5.5 W m^−2^ could be generated by a three‐parallel TENG, which made it able to light up 32 LEDs only by manual pressing. Moreover, Zhao et al. prepared a poly(2‐acrylamido‐2‐methyl‐1‐propanesulfonic acid)‐based conductive ionogel and applied it as both tribo‐positive layer and electrode (Figure 17f,g).^[^ ^120^ ^]^ The fabricated transparent and stretchable TENG was used as a self‐powered tactile sensor and acquired a maximum sensitivity of 1.76 V N^−1^, showing tremendous application potential in wearable and soft electronics. Also, Ding et al. for the first time reported the use of PEO as a tribo‐positive material for TENGs (Figure 17h).^[^ ^121^ ^]^ A 2 × 2 cm^2^ TENG made of spin‐coated flat PEO and PDMS films could yield outputs up to 970 V and 85 mA m^−2^ at a contact force of 50 N, which were much higher than those of the TENG using a nylon film as tribo‐positive layer. The results explicitly indicated that PEO has a higher positive tribo‐polarity even compared to nylon, possibly because of its lower work function and positively charged oxygen functional groups. That is not all, Kwak et al. demonstrated that butylated MF is also a promising tribo‐positive material (Figure 17i).^[^ ^123^ ^]^ By coupling the butylated MF with PTFE film, a rotation‐type TENG could produce root mean square outputs of 210 V and 24 mA m^−2^ at a rotation speed of 320 rpm. After AC‐to‐DC conversion and processing, these kinds of TENGs could be transformed into almost a DC power source with a constant output of 5 V and successfully charged a 700 µAh battery to 4 V in about 17 min.

## 3 PMs Adopted in Recently Developed Novel TENGs

Recently, a few fascinating and special TENGs using novel PMs for specific energy‐harvesting circumstances, such as access‐denied environments, in vivo, and liquid–liquid interactions, have been developed ever and again. In this part, we introduce several representative progresses and highlight the involved PMs, as well as potential applications of these TENGs.^[^ ^101^, ^124^, ^126^, ^127^, ^128^, ^129^, ^130^ ^]^

**Figure 18:**
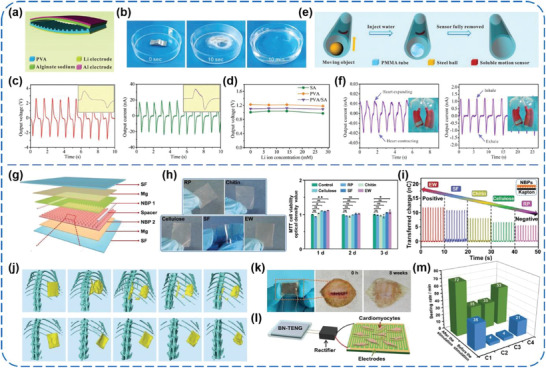
a–f) A recyclable and green TENG for energy harvesting in access‐denied environments. Reproduced with permission.^[^ ^124^ ^]^ Copyright 2017, Wiley‐VCH. g–m) Fully bioabsorbable natural‐materials‐based TENGs for in vivo applications. Reproduced with permission.^[^ ^126^ ^]^ Copyright 2018, Wiley‐VCH.

Liang et al. demonstrated a kind of fast soluble, recyclable, and green TENGs toward energy harvesting in access‐denied environments,^[^ ^124^ ^]^ which are based on cascade reactions and made of PVA and sodium alginate (SA) as triboelectric materials (**Figure** **18**a). Once contacted with water, the entire TENG can be fully dissolved and degraded into environmentally benign end products within minutes, as shown in Figure 18b. Driven by fingers, the TENG could produce outputs of 1.47 V, 3.9 nA, and 3.8 mW m^−2^. For application potential in access‐denied environments, the TENG‐enabled, self‐powered, clearable nanosensors for motion tracking (Figure 18e) and in vivo health monitoring (Figure 18f) were demonstrated by responding to external mechanical stimuli. More importantly, when the TENGs work beyond service life, they can be absolutely disintegrated and removed by water stream.

Jiang et al. developed a series of all‐natural‐materials‐based TENGs (BN‐TENGs) using five kinds of natural bioresorbable polymers (NBPs), including cellulose, rice paper (RP), egg white (EW), chitin, and silk fibroin (SF), for in vivo applications.^[^ ^126^ ^]^ As shown in Figure 18g, the fabricated BN‐TENGs consist of NBPs as triboelectric layers and spacers, magnesium (Mg) electrodes, and SF encapsulation layer. All these PMs stemmed from nature, such as wood, cotton, crab shells, wheat, egg, and so on. The decent viability of L929 cells proved that these NBPs are biocompatible and nontoxic in essence (Figure 18h). On the grounds of the control experiment, the electron‐donating ability of these NBPs was ranked as EW > SF > chitin > cellulose > RP (Figure 18i). As a result, with different pairwise combinations, a wide range of tunable outputs for *V*_OC_ from 8 to 55 V and *I*_SC_ from 0.08 to 0.6 µA could be achieved for the BN‐TENGs. In addition, the TENGs exhibited a fairly good biodegradability both in vitro and in vivo (in Sprague‐Dawley rats) and the degradation rate could be facilely regulated through modifying the SF encapsulation layers (Figure 18j,k). At last, a self‐powered stimulation system was built based on the BN‐TENG for effectively stimulating cardiomyocyte clusters (Figure 18l,m), which showed great potential for the BN‐TENGs in coordinating and repairing abnormal cardiomyocytes and myocardial tissues in vivo.

**Figure 19:**
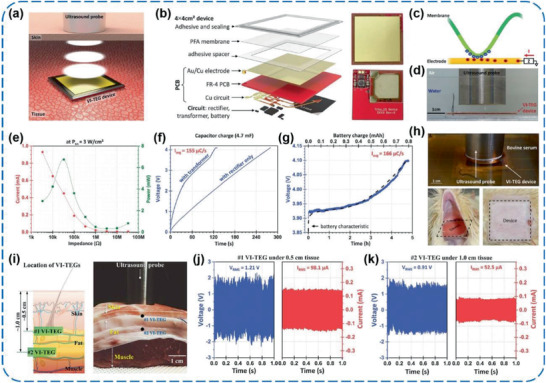
a–k) A high‐frequency vibrating and implantable TENG for harvesting ultrasound energy in vivo. Reproduced with permission.^[^ ^128^ ^]^ Copyright 2019, The American Association for the Advancement of Science.

More recently, Hinchet et al. reported a fantastic thin‐film type, high‐frequency vibrating, and implantable TEG (VI‐TEG) capable of harnessing transcutaneous ultrasound energy in vivo (**Figure** **19**a).^[^ ^128^ ^]^ The VI‐TEG works with a 50 µm thick perfluoroalkoxy (PFA) membrane as the tribo‐negative layer (Figure 19b), which is a copolymer of tetrafluoroethylene and perfluoroethers. Under ultrasound of 20 kHz and 3W cm^−2^ (Figure 19d), root mean square outputs of 9.71 V and 427 µA were generated by the VI‐TEG, combined with a maximum power density of 5.2 W m^−2^ at 33 kΩ (Figure 19e). The produced electric energy was able to charge a 4.7 mF capacitor and even a 700 µAh thin‐film Li‐ion battery (Figure 19f,g), which could further power several commercial implants. To simulate conditions closer to realistic medical applications, the performance of the VI‐TEG was explored in bovine serum, under skin of a rat, and even in a porcine tissue, respectively (Figure 19h,i). Under the ultrasound at 20 kHz and 1 W cm^−2^, an output power of ≈98.6 µW were generated (Figure 19j), which was still high enough to recharge the batteries of small implants (e.g., neurostimulators or pacemakers consuming ≈1–100 µW). This work builds a brand‐new concept and strategy for developing implantable power supplies towards transcutaneous electronic devices by using triboelectric technology, and it will be expected to vigorously promote the development of biocompatible and implantable PMs for in vivo TENGs.

**Figure 20:**
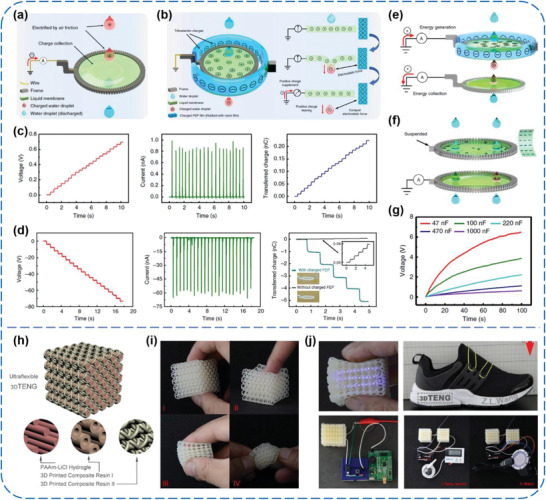
a–g) Power generation from the interaction of a liquid droplet and a liquid membrane. Reproduced with permission.^[^ ^129^ ^]^ Copyright 2019, Springer Nature. h‐j) Ultra‐flexible TENGs made by 3D printing technology for converting biomechanical energy form human motions. Reproduced with permission.^[^ ^130^ ^]^ Copyright 2018, Elsevier.

Apart from biocompatible and in vivo energy harvesting, Nie et al. showed an attractive approach for power generation from liquid–liquid (L‐L) interfacial interaction, which was realized by dripping liquid droplets through a freely suspended permeable liquid membrane.^[^ ^129^ ^]^ As schematically illustrated in **Figure** **20**a,b, two kinds of L‐L TENGs have been developed based on a grounded liquid membrane without charging, and a pre‐charged liquid membrane by surrounding an annular nylon‐rubbed FEP film, targeting the liquid droplets carrying with or without charges, respectively. The electric output performance and combined energy generation systems of the L‐L TENGs were systematically studied (Figure 20c–g). Here, it should be stressed that, although only PVA in the compositions of the liquid membrane belongs to PMs, this type of TENG still needs to remain put forward due to almost no advance appearing in the research filed of L‐L triboelectric power generation. In turn, this work might inspire us to pay more attention to and devote more effort to developing new liquid PMs possibly toward tribo‐energy conversion from L‐L interactions.

In addition, Chen et al. fabricated a practical, ultra‐flexible, and 3D TENG by a unique additive manufacturing technology, that is, hybrid 3D printing technique for the first time.^[^ ^130^ ^]^ Different from previously reported TENGs using common PM films, such 3D TENGs adopted acrylate‐based composite resins (with a high structural printing precision of 1 µm) as the triboelectric materials (Figure 20h). The excellent flexibility of the resins and PAAm‐LiCl hydrogels combined with the porous structure enable the 3D TENG to be easily compressed and twisted (Figure 20i). Optimized outputs of 10.98 W m^−3^ and 0.65 mC m^−3^ were obtained from the TENG with a size of 3.5 × 3.5 × 3.5 cm^3^ under a low compression frequency of 1.3 Hz. Various kinds of self‐powered systems were developed based on the TENGs as power supply, including a LEDs flickering and buzzing SOS distress signal system, LEDs lighting smart shoes, as well as a portable temperature sensor and a smart watch (Figure 20j). These demonstrations indicated great potential of the innovative 3D printing fabrication technology and 3D printable materials in TENGs technology.

## 4 Summary and Perspectives

In this review, frequently used and several newly developed PMs in currently reported TENGs are systematically summarized in detail (see in **Table** **1**). In fact, there are substantial efforts to improve TENGs’ performance through a series of methods based on PMs’ modification as well, mainly including tribosurface morphology engineering, surface molecular functionalization, and bulk composition modification.^[^ ^25^ ^]^ Since the performance of the TENGs depends heavily on the generated triboelectric charges during the friction process, it is essentially dominated by the nature of triboelectric materials, whether physicochemical properties or tribosurface morphology. Consequently, it is of great significance to ponder over how to substantially boost the TENGs’ performance to a higher level from the materials research point of view. Here, several key priorities of research challenges and directions for PMs’ development toward high‐performance TENGs are conceived to possibly give some inspiration and expected to be instructive to future research works.

1. The mechanisms how the different functional groups in PMs influence the charge transfer process are still unclear and scantly investigated. Therefore, it is necessary to study this microscopic process between different functional group pairs, aiming to see how the charge transfer occurs and particularly to explore what really contributes such a process in essence.
2. In addition to PMs’ modification, designing specific PMs pairs to possibly achieve some reversible reactions or phase transitions for enhancing charge transfer when contact‐separation occurs may be a fascinating research topic.
3. For some special application scenarios, novel PMs need to be developed and synthesized to construct high‐performance TENGs, such as energy harvesting in liquid conditions, in vivo, and even in access‐denied environments.
4. As a complementary or even indispensable branch of the energy field, TENGs technology has demonstrated tremendous potential toward low‐frequency wind or blue energy harvesting, which would have a far‐reaching influence on our future lives and even bring a great revolution in energy industry. In this regard, the machinability and scalability of PMs have to be further improved to make TENGs more adaptive and efficient for large‐scale wind and blue energy harvesting.
5. One usually concerns about the durability of TENGs with considering the softness of PMs. In fact, this is a misunderstanding about the triboelectrification (TE) and contact electrification (CE). The term of CE refers to a quantum mechanical electron/ion transfer process induced by a physical contact interaction between any two materials, in any states (i.e., solid, liquid, and gas), and in a wide environmental temperature range (up to ≈400 °C).^[^ ^131^ ^]^ Therefore, the materials would be electrically charged with opposite polarization after CE process. Such an effect is universal, ubiquitous, and fundamentally unique in nature. As for the term of TE, it is a convolution of CE and tribology processes, which means the mechanical rubbing between two materials, so that the TE and CE are inseparable.^[^ ^27^ ^]^ As such, it should be clear that TE is an engineering term, while CE is a science term. For TENGs, one only needs CE and rubbing of the two materials is unnecessary. In reality, the surface charges reach a saturation if the two materials being contacted for about 10 times and they remain on surfaces for hours before disappearing. Therefore, a physical contact is only needed at the beginning of the operation, and then the TENGs can operate for a few hours without physical contact of the two materials. This can be a guidance for designing long‐lifetime TENG.^[^ ^132^ ^]^

**Table 1:** A summary of frequently used PMs in common TENGs and several PMs adopted in some novel TENGs

Main PMs/Reference								
Tribo‐negative PMs				Tribo‐positive PMs				
PTFE^[^ ^35^, ^36^, ^37^, ^38^, ^39^, ^40^, ^41^, ^42^, ^43^, ^44^, ^45^, ^46^ ^]^	FEP^[^ ^47^, ^48^, ^49^, ^50^, ^51^ ^]^	PVDF^[^ ^57^, ^58^, ^59^, ^60^, ^61^, ^62^, ^63^, ^64^, ^65^ ^]^	PDMS^[^ ^54^, ^69^, ^70^, ^71^, ^72^, ^73^, ^74^, ^75^, ^76^, ^77^, ^78^ ^]^	Nylon^[^ ^45^, ^46^, ^102^, ^103^, ^104^, ^105^, ^106^, ^107^ ^]^	Cellulose^[^ ^110^, ^111^, ^112^, ^113^ ^]^	Silk^[^ ^114^, ^115^, ^116^ ^]^	TPU^[^ ^117^, ^118^ ^]^	Ppy^[^ ^119^ ^]^
PI^[^ ^82^, ^83^, ^84^, ^85^, ^86^, ^87^, ^88^ ^]^	PET^[^ ^89^, ^90^, ^91^, ^92^, ^93^, ^94^, ^95^, ^96^ ^]^	PMMA^[^ ^97^ ^]^	Gelatin^[^ ^98^ ^]^	PEO^[^ ^121^, ^122^ ^]^	Ionic gel^[^ ^120^ ^]^	PVA^[^ ^101^, ^124^ ^]^	MF^[^ ^123^ ^]^	Peptides^[^ ^125^ ^]^

PMs for several novel TENGs/References				
Soluble TENG	Bioabsorbable TENG	Implantable TENG	Permeable TENG	3D printable TENG
PVA^[^ ^124^ ^]^	EW/SF/chitin/cellulose/RP^[^ ^126^, ^127^ ^]^	PFA^[^ ^128^ ^]^	PVA^[^ ^129^ ^]^	Acrylate resins^[^ ^130^ ^]^

**Figure 21:**
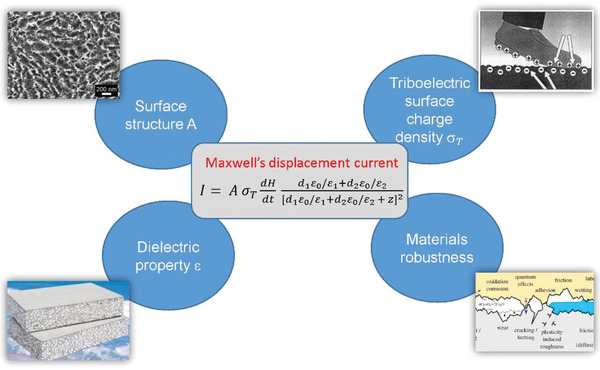
General guidance of choosing the PMs and related composites for high‐performance TENG considering surface structure, surface charge density, dielectric property, and surface robustness. Reproduced with permission.^[^ ^27^ ^]^ Copyright 2020, Elsevier.

All in all, what is the general guidance for developing the advanced PMs for TENGs? We use **Figure** **21** to illustrsate this problem. From the Maxwell's displacement current, the output of TENGs is related to materials’ permittivity, triboelectric charge density, and surface morphology. A composite containing possibly inorganic high permittivity materials may be needed in order to optimize the permittivity. Of course, the most important factor is how to enhance the surface charge density. For this purpose, development or modification of PMs at the molecular level for achieving better charge transfer or capturing capability is urgent. In addition, it may not be best if the surface morphology is too flat due to the limited contact between the two materials at atomic scale. A too rough surface would increase friction, but yet reduce the surface robustness at the same time, which may deteriorate the stability and lifetime of the TENGs. Therefore, the surface engineering of PMs is also a considerably critical point both for maximizing the contact area and the robustness as well as the lifetime of the TENGs. Given all these considerations, we anticipate that there will be a great opportunity for materials scientists and chemists to make efforts in tribo‐materials’ optimization for substantially enhancing the performance of TENGs.

## Conflict of Interest

The authors declare no conflict of interest.
